# Correlation between preoperative peripheral blood NLR, PLR, LMR and prognosis of patients with head and neck squamous cell carcinoma

**DOI:** 10.1186/s12885-023-11752-y

**Published:** 2023-12-18

**Authors:** Jiao Zhou, Sheng Wei, Xiumei Guo, Yanjun Huang, Yizheng Zhang, Yuming Hong, Xiaofang Chen, Ming Lu, Feng Zheng, Chaohui Zheng

**Affiliations:** 1 Department of Otolaryngology, The Second Affiliated Hospital of Fujian Medical University, Quanzhou, 362000 China; 2 Department of General Surgery, The Second Affiliated Hospital of Anhui Medical University, Hefei, 230000 China; 3 Department of Neurosurgery, The Second Affiliated Hospital of Fujian Medical University, Quanzhou, 362000 China

**Keywords:** Head and Neck squamous cell carcinoma, Neutrophil, Platelet, Lymphocyte, Monocyte, Prognosis

## Abstract

**Background:**

Markers that can be used to evaluate the prognosis of patients with head and neck squamous cell carcinoma (HNSCC) remain undefined.

**Objective:**

This study aimed to investigate the prognostic impact of preoperative neutrophil-to-lymphocyte ratio (NLR), platelet-to-lymphocyte ratio (PLR), and lymphocyte-to-monocyte ratio (LMR) in patients with HNSCC who underwent surgery-based treatment for the first time.

**Methods:**

This retrospective study included patients HNSCC who underwent surgery-based treatment at our institution between January 2018 and December 2020. Specificity and sensitivity were analyzed using receiver operating characteristic (ROC) curves and the critical value was determined. Patients were divided into low and high groups according to NLR, PLR, and LMR the critical value. Log-rank and Cox proportional hazards models were used to evaluate the associations between preoperative NLR, PLR, LMR, and overall survival (OS).

**Results:**

A total of 304 patients with HNSCC were included, of whom 190 (62.5%) and 114 (37.5%), 203 (66.8%) and 101 (33.2%), 98 (32.2%), and 206 (67.8%) cases were classified as low NLR and high NLR groups, low PLR and high PLR groups, and low LMR and high LMR groups, respectively. Univariate analysis showed that white blood cell count (WBC), neutrophil count (NEU), platelet count (PLT), NLR, pathologic N stage (pN stage), TNM stage and postoperative complications were significantly associated with OS (*p* < 0.05). Multivariate analysis showed that NEU, NLR, TNM stage and postoperative complications were independent negative prognostic factors for HNSCC (*p* < 0.05).

**Conclusion:**

Preoperative NLR is an independent negative prognostic factor for HNSCC. Patients with an increased NLR may have a poor OS.

## Background

Cancer-related mortality is a major cause of death worldwide, accounting for 13% of total human deaths [1]. Head and neck cancer is the sixth most common cancer globally, with more than 650,000 new cases and 350,000 deaths annually [1]. Head and neck squamous cell carcinoma (HNSCC) accounts for more than 90% of all malignant head and neck tumors. Its etiological factors are related mostly to tobacco, alcohol, viral pathogens, genetic factors, radiation, occupational exposure, and immune deficiency [2, 3]. The mainstay of treatment for HNSCC is surgery, supplemented with radiotherapy and chemotherapy. Despite recent advances in therapy, the long-term prognosis remains poor, with a 5-year survival rate of less than 50% [4]. Therefore, the evaluation of new prognostic markers is of great significance for predicting the survival of patients with HNSCC and for optimizing treatment strategies.

**Fig. 1:**
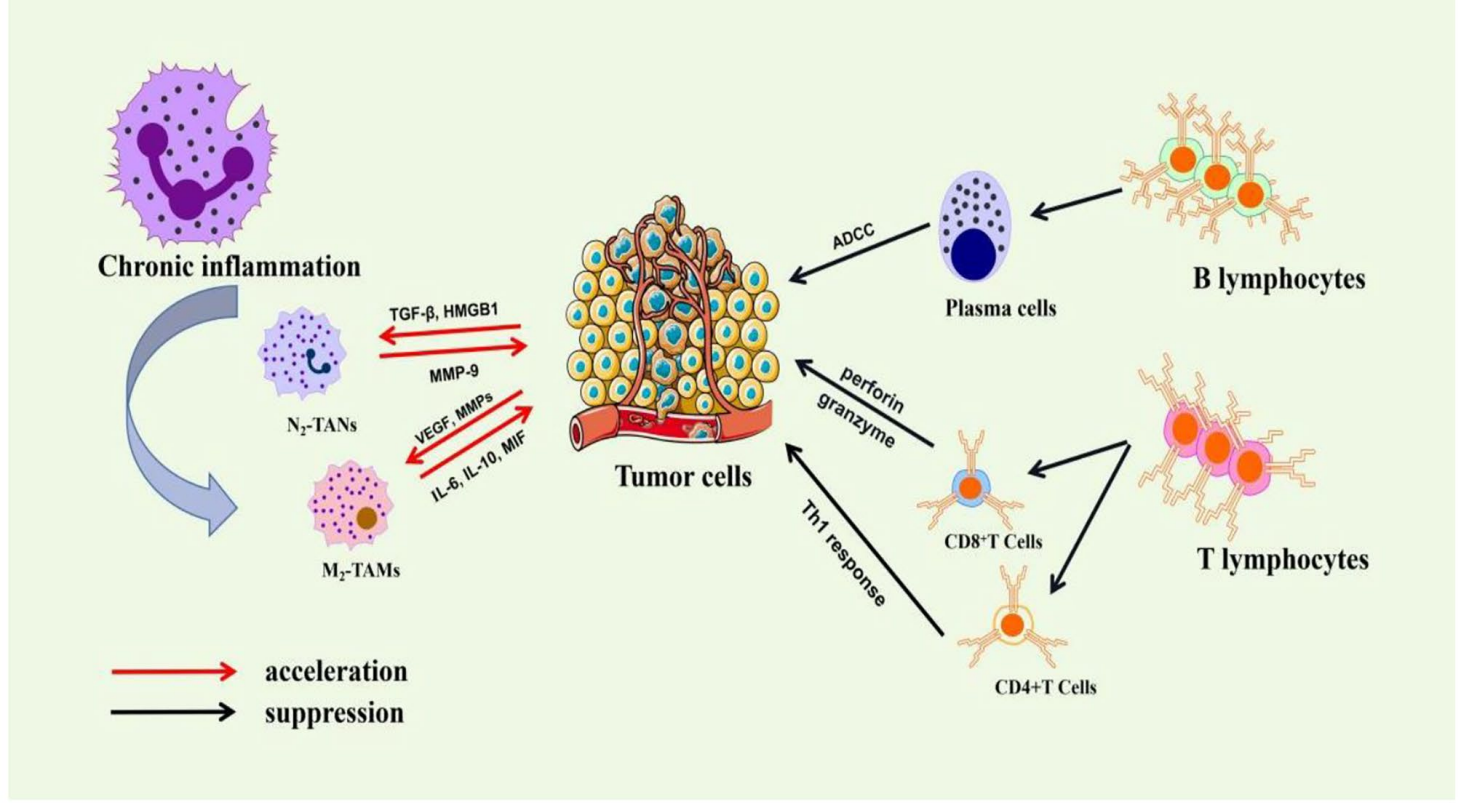
The relationship between chronic inflammation and cancer development. Tumor cells secret various cytokines, chemokines, and inflammatory mediators, recruiting a large number of immunosuppressive cells (e.g. M2-tumour-associated macrophages (M_2_-TAMs), N2-tumor-associated neutrophils (N_2_-TANs)). These immunosuppressive cells provide a rich proangiogenic and pro-tumoral microenvironment, and prevent the innate immunity and T-cell anti-tumor immunity. Immune cells (e.g. T lymphocytes, B lymphocytes) secrete cytokines and antibodies to play an anti-tumor role

Over the past decade, numerous studies have demonstrated a link between inflammation and cancer [5–8] (as shown in Fig. 1). The presence of leukocytes within tumors provided the first indication of a possible link between inflammation and cancer [9, 10]. Chronic inflammation can promote the occurrence and development of cancer by changing the microenvironment of tumor cells and activating endogenous or exogenous signals involving a variety of inflammatory mediators and proteins. It has been linked to various steps in tumorigenesis, including cellular transformation, promotion, survival, proliferation, invasion, angiogenesis, and metastasis [5].

The neutrophil-to-lymphocyte ratio (NLR) was first proposed by Bass et al. in 1983 [11], as an inflammatory index associated with tumorigenesis, invasiveness, and poor prognosis [12]. More recent studies have confirmed that NLR is closely related to the long-term prognosis of multiple solid malignancies [13–18]. In addition, platelet-to-lymphocyte ratio (PLR) and lymphocyte-to-monocyte ratio (LMR) have also been reported to be closely related to the long-term prognosis of multiple solid malignancies [19–21]. Recently, there have been several studies published on this topic, including a few meta-analyses or systematic reviews and studies limited to specific areas and treatment details. The prognostic variables of HNSCC reported in the literature included NLR, PLR, LMR, etc. However, there is so far no studies comparing the predictive value of NLR, PLR, and LMR for the overall survival prognosis of HNSCC. Therefore, we aimed to investigate the prognostic impact of preoperative NLR, PLR, and LMR on the overall survival (OS) of patients with HNSCC to optimize treatment strategies and improve the prognosis of patients with these tumors.

## Methods

### Patients

We retrospectively analyzed the data of patients with HNSCC who underwent surgery-based treatment for the first time at our institution between January 2018 and December 2020. The Inclusion criteria were as follows: (1) availability of complete clinical and pathological data including investigation, surgical, and pathological reports; (2) surgical treatment performed according to the National Comprehensive Cancer Network Guidelines (Version 1.2018) [22]; (3) postoperative pathological results confirming the diagnosis of HNSCC. The exclusion criteria were: (1) preoperative bacterial infection confirmed by biological tests including blood or sputum culture; (2) preoperative chemotherapy, radiotherapy, immunotherapy, or endocrine therapy; (3) distant metastasis confirmed by CT, MRI, or PET-CT imaging examinations before surgery; (4) non-squamous cell carcinoma confirmed by postoperative pathology; (5) nasopharyngeal carcinoma; (6) lost to follow-up; (7) concomitant malignant tumors or a history of malignant tumors; and (8) hematological diseases.

This study was conducted following the Code of Ethics of the World Medical Association (Declaration of Helsinki) for experiments involving humans, and the protocol was reviewed and approved by the MedicalEthics Committee of the Second Affiliated Hospital of Fujian Medical University (No. 6, 2023). All procedures performed in the study met the ethical standards of the Institutional Research Committee; and were in accordance with the relevant guidelines and regulations. Informed consent was obtained from all participants.

### Data extraction and follow-up

The patient’s basic clinical data were extracted from electronic medical records and included sex, age, smoking history, drinking history, and results of routine blood examination including white blood cell count (WBC), neutrophil count (NEU), lymphocyte count (LYM), monocyte count (MON), and platelet count (PLT), within one week before surgery, tumor site, TNM staging, pathologic N stage (pN stage), and postoperative complications. Clinicopathological data, including histopathology and surgical records, were retrieved from the patients’ medical records, and the follow-up time was set from the date of surgery to death, missed follow up, or December 31, 2021. The primary outcome was OS, defined as the time interval between the date of surgery and the date of death or last follow-up. Among them, the test reference values of our institute were WBC 4 ~ 10 × 10^^9^/L, NEU 1.80 ~ 6.30 × 10^^9^/L, LYM 1.10 ~ 3.20 × 10^^9^/L, PLT 100 ~ 350/L, and MON 0.12 ~ 0.8 × 10^^9^/L. Tumor staging was performed according to the guidelines for head and neck tumor TNM staging (eighth edition) formulated by the American Joint Commission on Cancer (AJCC) / Cancer /International Union Against Cancer (UICC) [20, 23].

### Data analysis

IBM SPSS (version 26.0;SPSS Inc., Chicago, Illinois, USA) and GraphPadPrism7 statistical software were used for data analysis. Measurement data were expressed as mean ± standard deviation (Mean ± SD) or median. Data were expressed as cases and percentages (%). Continuous variables were compared between groups using the t-test, and classified data were compared using the chi-square test. Specificity and sensitivity were analyzed using receiver operating characteristic (ROC) curves and the critical value was determined. And the continuity variables were converted into categorical variables. Patients were divided into low and high groups according to NLR, PLR, and LMR the critical value.The chi-square test was used to identify the correlation between the NLR, PLR, LMR, and clinical features. Spearman’s rank correlation analysis was used for correlation analysis among NLR, PLR, and LMR. A Cox regression model was used for proportional risk assessment in the univariate and multivariate analyses, and hazard ratios (HRs) and 95% confidence intervals (CIs) were used to report the magnitude of the differences and the strength of association. Statistical significance was set at *p* < 0.05.

## Results

### Baseline characteristics of patients

**Fig. 2:**
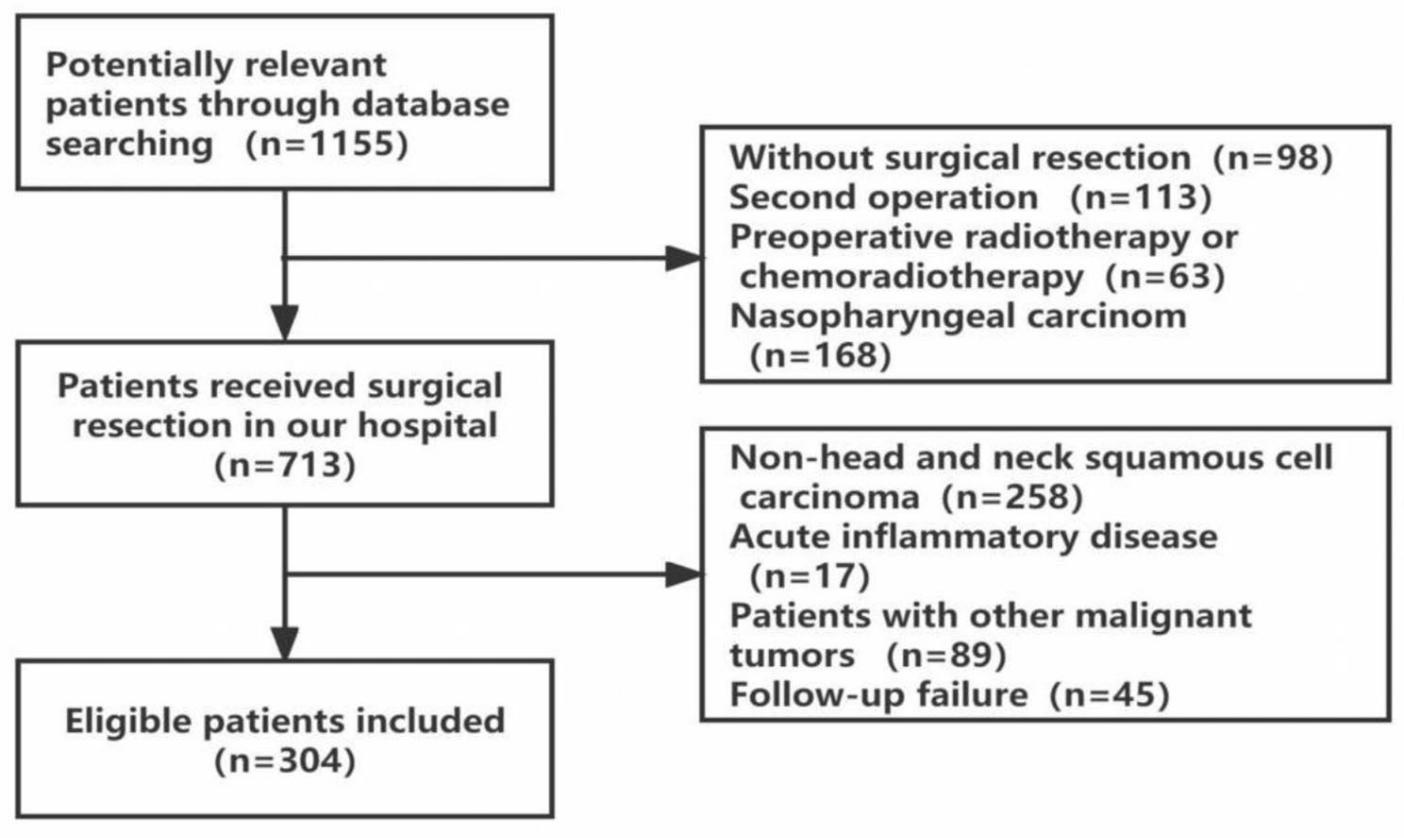
Flow chart of patient selection procedure

Between January 2018 and December 2021, 1155 patients with head and neck malignancies were admitted to our hospital. Among them, 98 had not undergone surgical resection, 113 underwent a second surgical treatment, 63 had preoperative radiotherapy or chemotherapy, 168 had nasopharyngeal carcinoma, 258 tumors were pathologically confirmed to be non-squamous cell carcinoma, 17 showed acute inflammation, 89 were accompanied by other malignant tumors and 45 did not complete follow up. Ultimately, 304 eligible patients were included in the final analysis (Fig. 2). Among them, 240 were males and 64 females, with a median age of 63 years (range: 28 ~ 91). The median follow-up time of the whole cohort was 24 months (range: 1–47), and the patients’ survival time was 25.20 ± 11.307 months (95% CI:23.92–26.47). The clinicopathological parameters included in this study aere summarized in Table 1.

**Table 1:** Clinicopathological data of 304 HNSCC patients

Variables	Patients (n = 304)
Gender(male/female)	240/64
Age (<63 ± 10.56/≥63 ± 10.56 years)	151/153
Smoking(yes/no)	190/114
Drinking(yes/no)	157/147
Tumor location (oral/oropharynx/larynx/hypopharynx/else)	116/29/119/23/17
T stage (T_1_/T_2_/T_3_/T_4_)	76/117/68/43
N stage (N_0_/N_1_/N_2_/N_3_)	216/34/54/0
M stage (M_0_/M_1_)	304/0
TNM stage (I/II/III/IV)	76/89/58/81
WBC counts(×10ˆ^9^/L)	6.885 ± 2.023
NEU counts(×10ˆ^9^/L)	4.747 ± 2.218
MON counts(×10ˆ^9^/L)	0.506 ± 0.213
LYM counts(×10ˆ^9^/L)	2.029 ± 0.843
PLT counts(×10ˆ^9^/L)	255.388 ± 74.927
NLR	2.684 ± 2.102
PLR	149.374 ± 85.085
LMR	4.448 ± 2.110
OS(months)	25.20 ± 11.307

T stage: tumor stage, N stage: lymph node stage, M stage: metastasis stage, TNM stage: tumor node metastasis stage, WBC: white blood cell, NEU: neutrophil, MON: monocyte, LYM: lymphocyte, PLT: platelet, NLR: neutrophil-to-lymphocyte ratio, PLR: platelet-to-lymphocyte ratio, LMR: lymphocyte-to-monocyte, OS: overall survival

### Critical value of prognostic markers

**Fig. 3:**
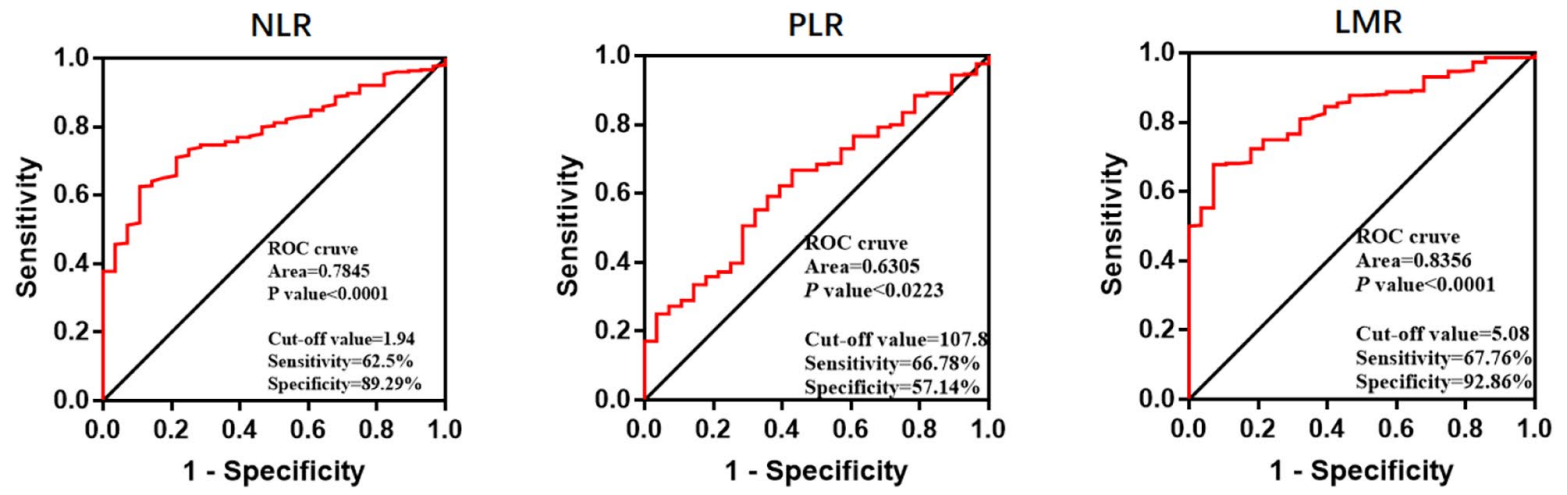
The ROC curve showing optimal NLR、PLR and LMR cut-off value

The optimal critical values of preoperative NLR, PLR, and LMR were 1.94, 107.8, and 5.08, respectively. Patients were divided into low and high groups according to NLR, PLR, and LMR the critical value. Low groups includes NLR < 1.94, PLR < 107.8, LMR < 5.08; High groups includes NLR ≥ 1.94, PLR ≥ 107.8, LMR ≥ 5.08. At this time, the area under the curve was 0.7845, 0.6305, and 0.8356; and the respective the levels of sensitivity were 62.5%, 66.78%, and 67.76%, while the levels of specificity were 89.29%, 57.14%, and 92.86%, respectively (Fig. 3). Correlation of baseline characteristics of different NLR, PLR and LMR groups in HNSCC patients. (Table 2).

**Table 2:** Correlation of baseline characteristics of different NLR, PLR and LMR groups in HNSCC patients

Variables	Parameters	NLR		χ^2^	P	PLR		χ^2^	P	LMR		χ^2^	P
		< 1.94	≥ 1.94			< 107.8	≥ 107.8			< 5.08	≥ 5.08		
Gender	Male	90	150	0	1	81	159	0.142	0.706	173(84)	67	9.74	0.002*
	Female	24	40			20	44			33	31		
Age (yr)	< 63 ± 10.56	42	73	0.076	0.783	40	75	0.203	0.653	79	36	0.074	0.786
	≥ 63 ± 10.56	72	117			61	128			127	62		
Smoking	Yes	66	124	1.651	0.199	64	126	0.048	0.826	135	55	2.51	0.113
	No	48	66			37	77			71	43		
Drinking	Yes	58	99	0.043	0.836	56	101	0.875	0.35	114	43	3.494	0.062
	No	56	91			45	102			92	55		
Tumor location	Oral	42	74		0.644	38	78		0.576	74	42		0.428
	Oropharynx	12	17			8	21			25	4		
	Larynx	42	77			39	80			77	42		
	Hypopharynx	13	10			11	12			16	7		
	Else	5	12			5	12			14	3		
pN stage	N_0_	31	57	0.273	0.601	27	61	0.361	0.548	59	29	0.029	0.864
	N_1 − 3_	83	133			74	142			147	69		
TNM stage	I-II	62	103	0.001	0.976	59	106	1.044	0.307	110	55	0.199	0.656
	III-IV	52	87			42	97			96	43		
Postoperative complications	Yes	14	39	3.365	0.067	14	39	1.341	0.247	32	21	1.603	0.205
	No	100	151			87	164			174	77		

NLR: neutrophil-lymphocyte ratio, PLR: platelet-lymphocyte ratio, LMR: lymphocyte-monocyte ratio, pN stage: pathological lymph node stage

*Statistically significant *p* < 0.05

In addition, Spearman’s rank correlation analysis was conducted among NLR, PLR and LMR, with the results showing that LMR is negatively correlated with NLR(ρ=- 0.519, *p* < 0.001) and PLR (ρ=- 0.496, *p* < 0.001), and NLR is positively correlated with PLR (ρ = 0.601, *p* < 0.001).

### Prognostic factors

Univariate analysis showed that leukocyte count (*p* = 0.03), NEU (*p* < 0.001), PLT count (*p* = 0.007), NLR (*p* < 0.001), pathologic N stage (pN stage) (*p* < 0.001), TNM stage (*p* < 0.001), and postoperative complications (*p* < 0.001) were significantly associated with OS of HNSCC patients. We included leukocyte count, NEU, PLT count, NLR, pN stage, TNM stage, and postoperative complications were included in multivariate analysis. However, multivariate analysis showed that NEU (hazard ratio [HR]:1.234, 95% CI:1.107–1.375, *p* < 0.001), NLR (HR:1.104, 95% CI:1.016-1.200, *p* = 0.019), TNM stage (HR:0.540, 95% CI:0.375–0.778, *p* = 0.001), and postoperative complications (HR:0.713, 95% CI:0.536–0.948, *p* = 0.020) were independent prognostic factors for OS in patients with HNSCC (Table 3).

**Table 3:** Comparison of overall survival for 304 HNSCC patients

Variables	Univariate analysis			Multivariate analysis		
	HR	95%CI	P *value*	HR	95%CI	P *value*
Gender(male/female)	1.442	0.708–2.940	0.313			
Age (< 63 ± 10.56/≥63 ± 10.56 years)	1.019	0.993–1.045	0.156			
Smoking(yes/no)	1.403	0.795–2.474	0.243			
Drinking(yes/no)	1.284	0.759–2.173	0.351			
Tumor location (oral/oropharynx/larynx/hypopharynx/else)			0.067			
WBC(< 7.96/≥7.96 × 10^9/L)	1.128	1.012–1.258	0.030*	0.914	0.800-1.044	0.185
NEU(< 4.57/≥4.57 × 10^9/L)	1.300	1.199–1.411	0.001*	1.234	1.107–1.375	0.000*
MON(< 0.48/≥0.48 × 10^9/L)	2.220	0.816–6.039	0.118			
LYM(< 2.44/≥2.44 × 10^9/L)	0.684	0.462–1.014	0.059			
PLT(< 246.5/≥246.5/L)	1.004	1.001–1.007	0.007*	1.002	0.998–1.005	0.327
NLR(< 1.94/≥1.94)	1.176	1.120–1.235	0.001*	1.104	1.016-1.200	0.019*
PLR(< 107.8/≥107.8)	1.002	1.000-1.005	0.073			
LMR(< 5.08/≥5.08)	0.930	0.815–1.062	0.285			
pN stage(N_0_/N_1 − 3_)	0.622	0.479–0.807	0.001*	1.097	0.799–1.508	0.567
TNM stage(I-II/III-IV)	0.474	0.351–0.642	0.001*	0.540	0.375–0.778	0.001*
Postoperative complication(yes/no)	0.607	0.460–0.799	0.001*	0.713	0.536–0.948	0.020*

Comparison of OS for HNSCC patients based on univariate and multivariate analysis in NLR, PLR and LMR. HR hazard ratio, CI confidence interval

*Statistically significant *p* < 0.05

**Fig. 4:**
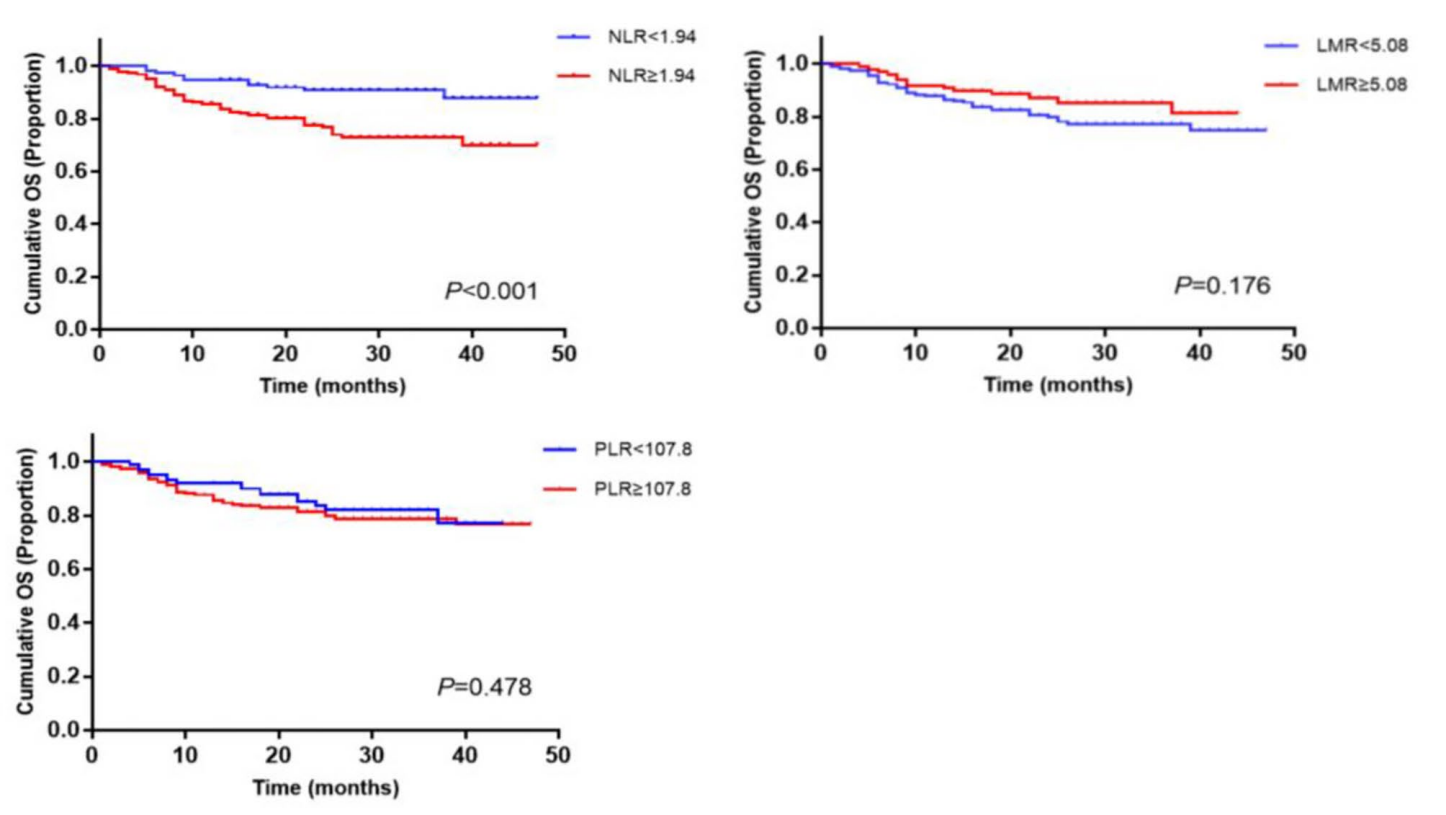
GraphPad Prism and Log-rank test showing overall survival curves of the NLR、PLR and LMR groups in 304 HNSCC patients

GraphPad Prism 7 was used and Log-rank test was performed to analyze the differences in OS between the low and the high NLR, PLR, LMR group. The results showed that OS rates of HNSCC patients in the low NLR group were significantly higher than that in the high NLR group (*p* < 0.001) (Fig. 4). The one-year overall survival rate of patients with NLR, PLR and LMR was not significantly different compared to the low and high groups, while the three-year overall survival rate was significantly different compared to the low and high NLR group. (Table 4).

**Table 4:** 1-year and 3-year OS rates of different NLR, PLR and LMR groups in HNSCC patients

Variables	1-Year survival rate(%)	3-Year survival rate(%)	*P* value
NLR < 1.94	103/114 = 90.4	32/37 = 86.5	*P* < 0.001*
NLR ≥ 1.94	144/190 = 75.8	35/50 = 70	
PLR < 107.8	84/101 = 83.2	19/27 = 70.4	*P* = 0.478
PLR ≥ 107.8	163/203 = 80.3	48/60 = 80	
LMR < 5.08	163/206 = 79.1	46/61 = 75.4	*P* = 0.176
LMR ≥ 5.08	84/98 = 85.7	21/26 = 80.8	

*Statistically significant *p* < 0.05

## Discussion

NLR has been extensively studied in many disciplines, including head and neck tumors [13–18]. Routine blood tests are highly repeatable, practical and can reflect systemic inflammation [24]. Inflammation is the interaction between various immune and inflammatory cells, chemokines, cytokines, and pro-inflammatory mediators [6]. It plays an important role in different stages of tumor occurrence, development, malignant transformation, invasion and metastasis [6]. Approximately 20% of cancers worldwide are associated with potential infections and inflammatory reactions [5]. Studies have revealed that there are molecular and cellular pathways between inflammation and cancer, which can be divided into two categories:(1) the external pathway: inflammation promotes the development of cancer; and (2) the intrinsic pathway: gene events that cause tumors to initiate the expression of inflammation-related process, thus guiding the generation of an inflammatory microenvironment [5, 25, 26].

In the present study, among patients with HNSCC who underwent surgery for the first time, an increase in NLR before surgery was associated with a poor prognosis, which is consistent with previous studies [27–29]. Our study also anand demonstrated that PLR and LMR are not suitable predictive indicators for the evaluation of survival and prognosis of patients with HNSCC; this is contrary to the findings of Takenaka and Yang et al. [30, 31]. This may be due to the following reasons. (1) In different studies, the selection of the critical value may have a certain impact on the results, which were calculated using ROC curves or medians [3]. In the present analysis, the ROC curve method was used; (2) the influence of coronary heart disease, hypertension, liver and kidney diseases, and other confounding factors on PLR and LMR [32–34].

According to the results of this study, increased NEU was an independent predictor of the prognosis of patients with HNSCC, which is consistent with the findings of Ross et al. [35]. Neutrophils account for 30 to70% of white blood cells in healthy adults and play an important role in cancer progression through various mechanisms, including promotion of immunosuppression, and cancer metastasis [31, 36]. The role of tumor-associated neutrophils includes: (1) inducing vascular endothelial growth factor, promoting angiogenesis, increasing tumor invasion, and weakening T lymphocytes resulting in promotion of tumor progression [37]; (2) reactive oxygen species production leading to damage of cellular DNA [5, 36]; and (3) secretion of various proteases, including neutrophil elastase, and matrix metalloproteinase-9. Neutrophils also play a role in promoting tumor development through mechanisms, including epithelial-to-mesenchymal transformation and extracellular matrix remodeling, leading to enhanced metastasis [36, 38]. In contrast, lymphocytes play an antitumor role by inducing cytotoxic cell death and inhibiting tumor cell proliferation and migration [39]. The interaction between these two leukocyte subtypes may explain the predictive value of the NLR.

Currently, factors impacting the prognosis of patients with malignant tumors include TNM stage, pathological N stage, and vascular or nerve invasion [40]. Although in the present study univariate analysis demonstrated that lymph node metastasis was significantly correlated with patient survival and prognosis, multivariate Cox regression model analysis showed that lymph node metastasis was not significantly correlated with patient survival or prognosis, which is inconsistent with the results analyzed by Ross D Dolan [35] et al. This may be due to the relatively small sample size of the present study. In our study, there were 304 patients with HNSCC, including 220 patients without lymph node metastasis, 31 of whom died, resulting in a mortality rate of 14.1%. Among the remaining 84 patients with lymph node metastasis 26 died, (mortality rate, 31%).

Our study had some limitations. First, this was a single-center retrospective analysis, and there may have been a selection bias and confounding variables. In the present study, 17 patients (5.6%) with other types of HNSCC, including nasal, lip, and maxillary sinus cancers were included, which may have introduced some heterogeneity. Second, this study lacks data on disease-free survival (DFS), although OS is considered as the standard indicator of cancer prognosis. Third, there is no uniform standard for the selection of the best cutoff value of hematological indicators, which may have biased the results. In addition, postoperative complications include those of various degrees of severity, and it would be better to analyze them separately into minor and major, or to target major. However, as this study is under retrospective design, postoperative complications were recorded only in types and incidence. Further analysis based on severity classification therefore can not be achieved in the present study. Additional attention should be paid on this issue. Finally, owing to the unavailability data, further analysis of disease-specific survival could not be performed in the present study. Future studies should focus on this topic.

## Conclusions

Preoperative NLR is an independent prognostic factor for HNSCC. Patients with an increased NLR may have poor OS. Future studies with larger sample sizes, preferably using a multicenter prospective design are warranted to confirm our findings.

## Data Availability

The datasets used and analysed during the current study available from the corresponding author on reasonable request.

## Abbreviations

NLR: Neutrophil-to-lymphocyte ratio
PLR: Platelet-to-lymphocyte ratio
LMR: Lymphocyte-to-monocyte ratio
HNSCC: Head and neck squamous cell carcinoma
OS: Overall survival
WBC: White blood cell count
NEU: Neutrophil count
PLT: Platelet count
LYM: Lymphocyte count
MON: Monocyte count
pN: Stage pathologic N stage
TNM: Stage tumor node metastasis stage
M2-TAMs: M2-tumour-associated macrophages
N2-TANs: N2-tumor-associated neutrophils
ROC: Receiver operating characteristic
CI: Confidence interval
HR: Hazard ratio
